# Induction of labour versus expectant management for nulliparous women over 35 years of age: a multi-centre prospective, randomised controlled trial

**DOI:** 10.1186/1471-2393-12-145

**Published:** 2012-12-11

**Authors:** Kate F Walker, George Bugg, Marion Macpherson, Carol McCormick, Chris Wildsmith, Gordon Smith, Jim Thornton

**Affiliations:** 1 Nottingham University Hospitals NHS Trust, Nottingham, UK; 2 Trustee for SANDS, London, UK; 3 University of Cambridge, Cambridge, UK; 4 University of Nottingham, Nottingham, UK

**Keywords:** Induction of labour, Advanced maternal age, Perinatal outcome, Caesarean delivery

## Abstract

**Background:**

British women are increasingly delaying childbirth. The proportion giving birth over the age of 35 rose from 12% in 1996 to 20% in 2006. Women over this age are at a higher risk of perinatal death, and antepartum stillbirth accounts for 61% of all such deaths. Women over 40 years old have a similar stillbirth risk at 39 weeks as women who are between 25 and 29 years old have at 41 weeks.

Many obstetricians respond to this by suggesting labour induction at term to forestall some of the risk. In a national survey of obstetricians 37% already induce women aged 40–44 years. A substantial minority of parents support such a policy, but others do not on the grounds that it might increase the risk of Caesarean section. However trials of induction in other high-risk scenarios have not shown any increase in Caesarean sections, rather the reverse. If induction for women over 35 did not increase Caesareans, or even reduced them, it would plausibly improve perinatal outcome and be an acceptable intervention. We therefore plan to perform a trial to test the effect of such an induction policy on Caesarean section rates.

This trial is funded by the NHS Research for Patient Benefit (RfPB) Programme.

**Design:**

The 35/39 trial is a multi-centre, prospective, randomised controlled trial. It is being run in twenty UK centres and we aim to recruit 630 nulliparous women (315 per group) aged over 35 years of age, over two years. Women will be randomly allocated to one of two groups:

Induction of labour between 39^0/7^ and 39^6/7^ weeks gestation.

Expectant management i.e. awaiting spontaneous onset of labour unless a situation develops necessitating either induction of labour or Caesarean Section.

The primary purpose of this trial is to establish what effect a policy of induction of labour at 39 weeks for nulliparous women of advanced maternal age has on the rate of Caesarean section deliveries. The secondary aim is to act as a pilot study for a trial to answer the question, does induction of labour in this group of women improve perinatal outcomes? Randomisation will occur at 36^0/7^ – 39^6/7^ weeks gestation via a computerised randomisation programme at the Clinical Trials Unit, University of Nottingham. There will be no blinding to treatment allocation.

**Discussion:**

The 35/39 trial is powered to detect an effect of induction of labour on the risk of caesarean section, it is underpowered to determine whether it improves perinatal outcome. The current study will also act as a pilot for a larger study to address this question.

**Trial registration:**

ISRCTN11517275

## Background

The average age at childbirth in the UK is increasing, and more women are giving birth over the age of 35 years [1]. In 1996, 12% of live births were to women over the age of 35 years. By 2006 that figure had risen to 20%. The Office of National Statistics estimate that in 2006, 5.6% of live births were to nulliparous women over the age of 35 years. In Scotland, the figure in 2005 was approximately 10% [2].

Women over 35 years are at higher risk of antepartum and intrapartum stillbirths and neonatal deaths, of hypertensive disorders, gestational diabetes, placenta praevia and placental abruption [3-5]. They are at increased risk of preterm labour and of bearing macrosomic (>3999 g) or low birth weight infants (<2500 g). The women themselves typically believe that their age puts their infant at increased risk [4]. Unsurprisingly they have higher rates of obstetric intervention.

The Caesarean section rate for nulliparous women over 35 years is 38% and 50% in women over 40 years [4]. In nulliparous women, the relationship between maternal age and delivery by emergency Caesarean is linear which suggests a biological effect of advancing maternal age on labour performance, rather than simply obstetrician or maternal preference [2].

Antepartum stillbirth is particularly important in this group of women, because they are relatively unlikely to have future pregnancies. Induction at or before term is logical because, although the perinatal mortality rate is lowest at 41 weeks, the gestational age associated with the lowest cumulative risk of perinatal death is 38 weeks [6]. This is because there is a cumulative risk of stillbirth in all of the weeks of gestation leading up to the eventual week of delivery. The largest increase in risk of stillbirth for women over 35 years of age starts at 39 weeks and peaks at 41 weeks. Women over 40 years old have a similar stillbirth risk at 39 weeks as women aged 25–29 years old have at 41 weeks. Once they pass 40 weeks gestation their risk of stillbirth exceeds that of all women <40 years old at term [7]. Nulliparous women have a higher risk of stillbirth than multiparous women for all maternal age groups. Obstetricians and parents generally understand this, but may avoid routine induction because of its association with Caesarean section.

Emergency Caesarean sections carry an increased maternal morbidity and mortality compared to vaginal delivery and have consequences for future pregnancies (uterine rupture and placenta praevia). Caesarean sections cost £760 more than a vaginal delivery [8]. A recent trial of induction of labour at term for women identified as high risk for emergency CS (higher the risk score, earlier the induction), found in the treatment group a similar CS rate, a higher vaginal birth rate and a reduced NICU admission and adverse perinatal outcome rate [9].

Perinatal deaths affect 0.8% of all pregnancies to women over the age of 35 years, and 1% of all pregnancies to women over the age of 40 years [3]. The distribution of maternal age among the mothers having perinatal deaths is skewed towards advanced maternal age compared with the general maternity population. Mothers having stillbirths and neonatal deaths are more likely to be older (40+). When an adverse event is rare, a large sample size is needed for a trial to prove that an intervention reduces the risk of that adverse event occurring.

Many obstetricians already induce older pregnant women at term (39% women aged 40–44, 58% women aged over 45), and many others believe that induction would improve perinatal outcomes but are reluctant to offer it for fear of increasing Caesarean rates [10]. However, it is equally plausible that induction might reduce Caesarean section in which case an effective intervention is being under implemented.

There is a growing body of evidence that induction of labour at term does not increase emergency Caesarean section rates and does not increase intrapartum deaths. In two other large multi-centre RCTs where induction of labour was compared with expectant management for term prelabour rupture of membranes and for post-term pregnancies, a policy of induction did not increase the Caesarean section rate [11,12]. More recently a large randomised controlled trial comparing induction of labour (between 36 and 41 weeks) versus expectant management for women with gestational hypertension or mild pre-eclampsia found that the rate of Caesarean delivery was the same in both groups [13]. Induction versus expectant monitoring for intrauterine growth restriction at term, a multi-centre RCT of 650 women found no difference in CS rates between the two groups [14].

Unfortunately there is no hard evidence to guide policy on induction of labour for advanced maternal age.

## Design

### Type of study

A multi-centre prospective randomised controlled trial.

Duration: 24 months

### Number and type of participants

630 nulliparous pregnant women over 35 years of age (315 per group).

### Recruitment and consent

Women will be identified in the antenatal period by their obstetrician or midwife. They will be offered an information sheet. They will be offered trial entry, and if they agree will sign a written consent. Women who are aged between 35–39 years at some of the participating centres will be community led care. Those women will be sent an invitation flyer or leaflet with their dating scan appointment to inform them of the trial and approached in person at their 20/40 anomaly scan at the hospital. The research midwives/principal investigator will see those women who express an interest in joining the trial at 36/40 for randomisation.

### Randomisation

Participants will be assigned to one of two treatment groups via a computerised randomisation programme at the Clinical Trials Unit, Nottingham University Hospitals NHS Trust. Randomisation will occur at 36^0/7^ – 39^6/7^ weeks gestation.

### Interventions

Women will be randomly allocated to one of two groups

• Treatment group: Women over the age of 35 years with a singleton live fetus in a cephalic presentation will be assigned to induction of labour between 39^0/7^ and 39^6/7^ weeks gestation.

• Control group: Women over the age of 35 years with a singleton live fetus in a cephalic presentation will be assigned to expectant management i.e. awaiting spontaneous onset of labour unless a situation develops necessitating either induction of labour or Caesarean Section. Those without any medical indication for induction will be offered induction of labour anywhere between T^+7^ and T^+14^, the exact time to be determined by consultant’s usual practice. No additional monitoring in the expectant management group prior to T^+14^ should be offered unless it is the consultant’s usual practice. If the patient declines induction of labour at T^+14^ the patient will be offered a scan for growth and liquor volume and offered alternate day or daily CTG monitoring depending on the consultants usual practice.

This is a pragmatic clinical trial where individual units will be able to follow their own policies for induction of labour. Each unit will record their local prostaglandin and oxytocin regime and Bishop score cut-off for amniotomy, prior to entry to the trial. Once this is recorded staff should as far as possible use the same induction protocol for all participants and also for those women who for whatever reason require induction in the “await spontaneous labour” group.

### Outcome measures

The primary end point is Caesarean section.

Secondary end points will include:

a) **Maternal outcomes**

Mode of delivery

Vaginal delivery

Assisted vaginal delivery (forceps or ventouse)

Caesarean section

Onset of labour

Spontaneous

Induction

Planned Caesarean section

Not in labour - emergency Caesarean section

Indication for induction of labour

Randomised to treatment

Gestational age > 41 weeks

Preterm (< 37 weeks) prelabour rupture of membranes

Term (> 37 weeks) prelabour rupture of membranes > 24 hours

Fetal growth restriction

Reduced fetal movements

Intrauterine fetal death

Pregnancy induced hypertension

Pre-eclampsia

Eclampsia

Obstetric cholestasis

Gestational diabetes

Suspected fetal distress

Maternal request

Other (free text)

*Method of induction of labour* (*as many as apply*)

Prostaglandin tablet regime

Prostaglandin gel regime

Prostaglandin slow-release pessary

Artificial rupture of amniotic membranes

Oxytocin (please do not tick if used to accelerate normal labour)

Indication for Caesarean section

Arrest of first stage of labour

Arrest of second stage of labour

Failed instrumental delivery

Fetal distress

Maternal complication

Elective

Other (free text)

Intrapartum complications

Placental abruption

Cord prolapse

Postpartum haemorrhage

Shoulder dystocia

Postpartum morbidity

Requiring blood transfusion

Systemic infection – temp > 38°C

b) **Neonatal outcomes**

Live birth

Stillbirth (a baby delivered with no signs of life after 24 completed weeks of pregnancy)

Birth weight

Sex

Death before discharge from hospital

Apgar at 1 min

Apgar at 5 min

Apgar at 10 minutes – allow missing data

Cord blood artery pH and BD – allow missing data

Cord blood vein pH and BD – allow missing data

NICU admission – duration (days)

If no cord blood and NICU admission required – first fetal pH obtained

Birth trauma

Subdural haematoma

Intracerebral or intraventricular haemorrhage

Spinal-cord injury

Basal skull fracture

Peripheral-nerve injury present at discharge from hospital

Long bone fracture

Seizures (occurring at less than 24 hr of age or requiring two or more drugs to control them)

Hypotonia (for at least 2 hrs)

Abnormal level of consciousness (hyperalert, drowsy or lethargic; stupor/decreased response to pain; coma)

Tube feeding for > 4 days

Intubation and ventilation for > 24 hrs

Cooling required

Oxygen required

CPAP required

c) **Outcomes for pilot study**

The recruitment rate per hospital.

The age distribution of participating women.

Compliance with the treatment arms of the trial.

The overall gestational age distribution of the two groups.

Completeness of outcome data.

d) **Maternal delivery expectation**/**experience** measured by the Childbirth Experience Questionnaire [15]. We will invite a sample of participants to join focus groups to explore in-depth their views.

### Participant entry

#### Pre-randomisation evaluations

Blood pressure and urinalysis to exclude proteinuric hypertension.

Estimated date of delivery based on a dating scan performed before 22 weeks gestation.

#### Inclusion criteria

Nulliparous women who will be over 35 years at the expected date of delivery, with:

• A singleton live fetus.

• A cephalic presentation.

• Gestational age between 36^0/7^ and 39^6/7^.

• No medical contra-indication to induction of labour.

• No medical contra-indication to pregnancy being. allowed to proceed to term plus 10 days.

• Willingness to participate in the trial.

• Written informed consent.

#### Exclusion criteria

Women with a known lethal fetal congenital abnormality.

Women with a contraindication to labour or vaginal delivery (e.g. evidence of fetal compromise such that labour would be contraindicated; fetal congenital anomaly or condition that might cause a mechanical problem at delivery such as hydrocephalus or cystic hygroma; placenta praevia).

Women with a contraindication to expectant management (e.g. gestational diabetes, proteinuric hypertension (24 hr urine collection protein >250 mg/l or BP > 140/90 on more than two occasions, two hrs apart).

Women with a previous myomectomy.

Women who book late for antenatal care and have no dating scan performed before 22 weeks to provide an accurate EDD.

Women who have undergone IVF using donor eggs in the current pregnancy.

#### Withdrawal criteria

There are no formal stopping rules. The Data Monitoring and Ethics Committee will review un-blinded results, adverse events and any other published data at least annually, and may advise stopping if there is clear evidence of benefit or harm in one or other group.

#### Adverse events

##### Definitions

**Adverse Event (AE):** any untoward medical occurrence in a patient or clinical study subject.

**Serious Adverse Event (SAE):** any untoward and unexpected medical occurrence or effect that:

• Results in death.

 • Is life-threatening – *refers to an event in which the subject was at risk of death at the time of the event*; *it does not refer to an event which hypothetically might have caused death if it were more severe.*

• Requires hospitalisation, or prolongation of existing inpatients’ hospitalisation.

• Results in persistent or significant disability or incapacity.

• Is a congenital anomaly or birth defect.

Medical judgement should be exercised in deciding whether an AE is serious in other situations. Important AEs that are not immediately life-threatening or do not result in death or hospitalisation but may jeopardise the subject or may require intervention to prevent one of the other outcomes listed in the definition above, should also be considered serious.

#### Reporting procedures

All adverse events should be reported. Depending on the nature of the event the reporting procedures below should be followed. Any questions concerning adverse event reporting should be directed to the Chief Investigator in the first instance.

#### Non-serious AEs

All such events, whether expected or not, should be recorded.

#### Serious AEs

An SAE form should be completed and faxed to the Chief Investigator within 24 hours. However, adverse events do not include admissions or day unit attendances for fetal monitoring, maternal hypertension, antepartum haemorrhage, preterm labour, preterm prelabour rupture of membranes, abdominal pain, transverse or oblique lie or placenta praevia. Likewise hospitalisation for labour, normal delivery, Caesarean or for induction of labour, and hospitalisations for elective treatment of a pre-existing condition do not need reporting as adverse events. Admissions for common postpartum complications such as maternal hypertension, perineal problems, mental health problems, urinary problems, or infections do not need reporting as adverse events.

All SAEs should be reported to the Derby 1 REC where in the opinion of the Chief Investigator, the event was:

• ‘related’, i.e. resulted from the administration of any of the research procedures; and

• ‘unexpected’, i.e. an event that is not listed in the protocol as an expected occurrence.

Reports of related and unexpected SAEs should be submitted within 15 days of the Chief Investigator becoming aware of the event, using the NRES SAE form for non-IMP studies.

Local investigators should report any SAEs as required by their Local Research Ethics Committee and/or Research & Development Office.

Sponsor Contact Details for SAEs:

1. Email to rdsae@nhs.nuh.uk,

2. Fax to: 0115 8493295 and phone Research & Innovations Dept on 0115 9709049.

#### Assessment and follow-up

Post-randomisation data will be collected at discharge.

Local centres will identify an individual within their unit to collect outcome data at hospital discharge from the hospital notes. Outcome data will be collected immediately following discharge by the same individual identified above.

Part of the outcome data will include a maternal expectation/experience questionnaire to be completed by mothers one month following delivery [15].

Participating women who deliver at NUH will also be invited to join a focus group to explore in-depth their views. The information obtained from the focus groups will allow us to collect richer outcome data from the trial that will allow a more in-depth contextual appreciation of the participants experience than a prescriptive survey would allow. The focus groups will be conducted by Kate Walker supervised by Associate Professor in Midwifery Denis Walsh who has agreed to be a co-supervisor on her PhD and will take place within 8 to 12 weeks following birth. This allows for women to reflect upon their birth as well as recover from it.

The focus groups will be split into two subgroups: women who underwent induction of labour and women who were part of the non-intervention group who underwent expectant management.

We will aim for 6 women per group.

The focus groups aim to provide a comprehensive sense of the acceptability of this treatment option to women.

The trial will end once all data has been collected for the 630 participants or at 2 years whichever is the later time point.

#### Statistics and data analysis

##### Sample size and justification

CS rates from 2004–2008 for women with singleton pregnancies in labour at term, excluding breech presentation was approximately 22% among women 35–39 years of age and approximately 27% among women aged 40 years or older (Smith GCS, personal communication).

Therefore assuming a Caesarean section rate of 25% in controls. This sample size has 80% power with a two-sided significance level of 5% to test the hypothesis that induction of labour reduces the Caesarean section rate to 16%, a 36% relative reduction (or a 9% absolute reduction).

We will recruit 315 women per group, a total of 630 women.

#### Statistical analysis

Demographic, measures of compliance and any other baseline data will be summarised by descriptive statistics (N (%), mean and standard deviation [sd], median, upper and lower quartiles {iqr}, minimum and maximum) or frequency tables, stratified by the two arms.

Effectiveness will be assessed using a generalised linear model (GLM) with the rate of Caesarean section as response and terms for treatment arm and centre. We will use the GLM to calculate relative risk and 95% confidence intervals. The primary analysis will be adjusted for recruitment centre.

Further details of the statistical analysis will be supplied in the Statistical Analysis Plan, to be finalised in a separate document before the data lock.

The analysis will be conducted according to the intention to treat (ITT).

Data and all appropriate documentation will be stored for a minimum of 5 years after the completion of the study, including the follow-up period.

#### Regulatory issues

##### Ethics approval

The Chief Investigator has obtained approval from the Derby 1 Research Ethics Committee. Where applicable the study must be submitted for Site Specific Assessment (SSA) at each participating NHS Trust. The Chief Investigator will require a copy of the SSA approval letter before accepting participants into the study. The study will be conducted in accordance with the recommendations for physicians involved in research on human subjects adopted by the 18th World Medical Assembly, Helsinki 1964 and later revisions.

#### Consent

Consent to enter the study must be sought from each participant only after a full explanation has been given, an information leaflet offered and time allowed for consideration. Signed participant consent should be obtained. The right of the participant to refuse to participate without giving reasons must be respected. After the participant has entered the study the clinician remains free to give alternative treatment to that specified in the protocol at any stage if he/she feels it is in the participant’s best interest, but the reasons for doing so should be recorded. In these cases the participants remain within the study for the purposes of follow-up and data analysis. All participants are free to withdraw at any time from the protocol treatment without giving reasons and without prejudicing further treatment.

#### Confidentiality

The Chief Investigator will preserve the confidentiality of participants taking part in the study and is registered under the Data Protection Act.

#### Indemnity

Standard NHS Indemnity applies.

#### Sponsor

Nottingham University Hospitals NHS Trust will act as the main sponsor for this study. Delegated responsibilities will be assigned to the NHS trusts taking part in this study.

#### Funding

The National Institute for Health Research – Research for Patient Benefit Programme is funding this study.

#### Audits

The study may be subject to inspection and audit by Nottingham University Hospitals under their remit as sponsor and other regulatory bodies to ensure adherence to GCP and the NHS Research Governance Framework for Health and Social Care (2^nd^ edition).

#### Study management

The day-to-day management of the study will be co-ordinated through the Trial Manager – Dr Kate Walker supported by the Trial Management Group (Professor Jim Thornton, Mr George Bugg, Miss Marion Macpherson, Professor Gordon Smith, Miss Carol McCormick, Mrs Nicky Grace and Mr Chris Wildsmith).

The Trial Steering Committee (TSC) will provide overall supervision of the trial including trial progress, adherence to the protocol, patient safety and consideration of new information arising during the period of recruitment to the trial.

The Data Monitoring and Ethics Committee (DMEC) will meet regularly to review un-blinded data.

#### Publication policy

All papers will be authored by the “35/39” trial study group. All contributors will be fully acknowledged.

## Abbreviations

AE: Adverse events; BD: Base deficit; BP: Blood pressure; CS: Caesarean section; CPAP: Continuous positive airway pressure; CTG: Cardiotocography; DMEC: Data monitoring and ethics committee; EDD: Expected date of delivery; GLM: Generalised linear model; GCP: Good clinical practice; IVF: In vitro fertilisation; NHS: National health service; NICU: Neonatal intensive care unit; NUH: Nottingham university hospitals NHS trust; REC: Research ethics committee; RCT: Randomised controlled trial; SAE: Serious adverse events; TSC: Trial steering committee.

## Competing interests

The authors declare that they have no competing interests.

## Authors’ contributions

All the authors have made substantial contributions to conception and design of the protocol. They have been involved in drafting the manuscript and revising it critically for important intellectual content. They have given final approval of the version to be published.

## Pre-publication history

The pre-publication history for this paper can be accessed here:

http://www.biomedcentral.com/1471-2393/12/145/prepub
